# Ponce De Leon Spring

**Published:** 1873-07

**Authors:** 


*Ponce de Leon Spring.*

We are authorized to state that the hotel at this famous spring will soon opened for the reception of visitors. Invalids will have all the comforts of a good hotel, witn suitable medical attention in connection with the almost marvelous virtues of the waters of this celebrated spring.

